# Supplementary material for: Microbial volatile communication in human organotypic lung models
Source: Nat Commun. 2017 Nov 24;8:1770. doi: 10.1038/s41467-017-01985-4 (PMC5701243; doi:10.1038/s41467-017-01985-4)
Supplement: Supplementary file 1 — Supplementary Information [file 41467_2017_1985_MOESM1_ESM.pdf]

a b

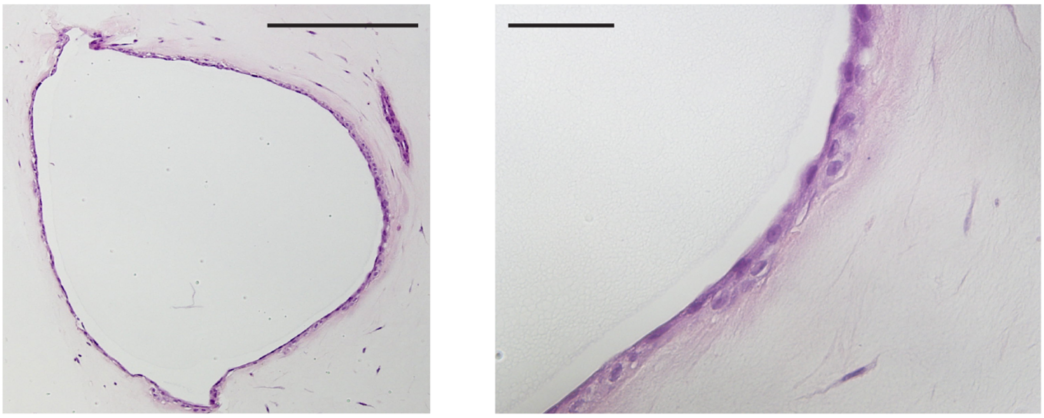

Supplementary Figure 1. Organotypic bronchiole histology. Models were removed from the PDMS molds after 7 days in culture, filled with agarose, histologically processed using Ventana products, and stained with H&E. (a) Center lumen image with surrounding fibroblasts. Scale bar is 250 μm. (b) Magnified image of the bronchial epithelial cells lining the center lumen and surrounding fibroblasts. Scale bar is 50 μm.

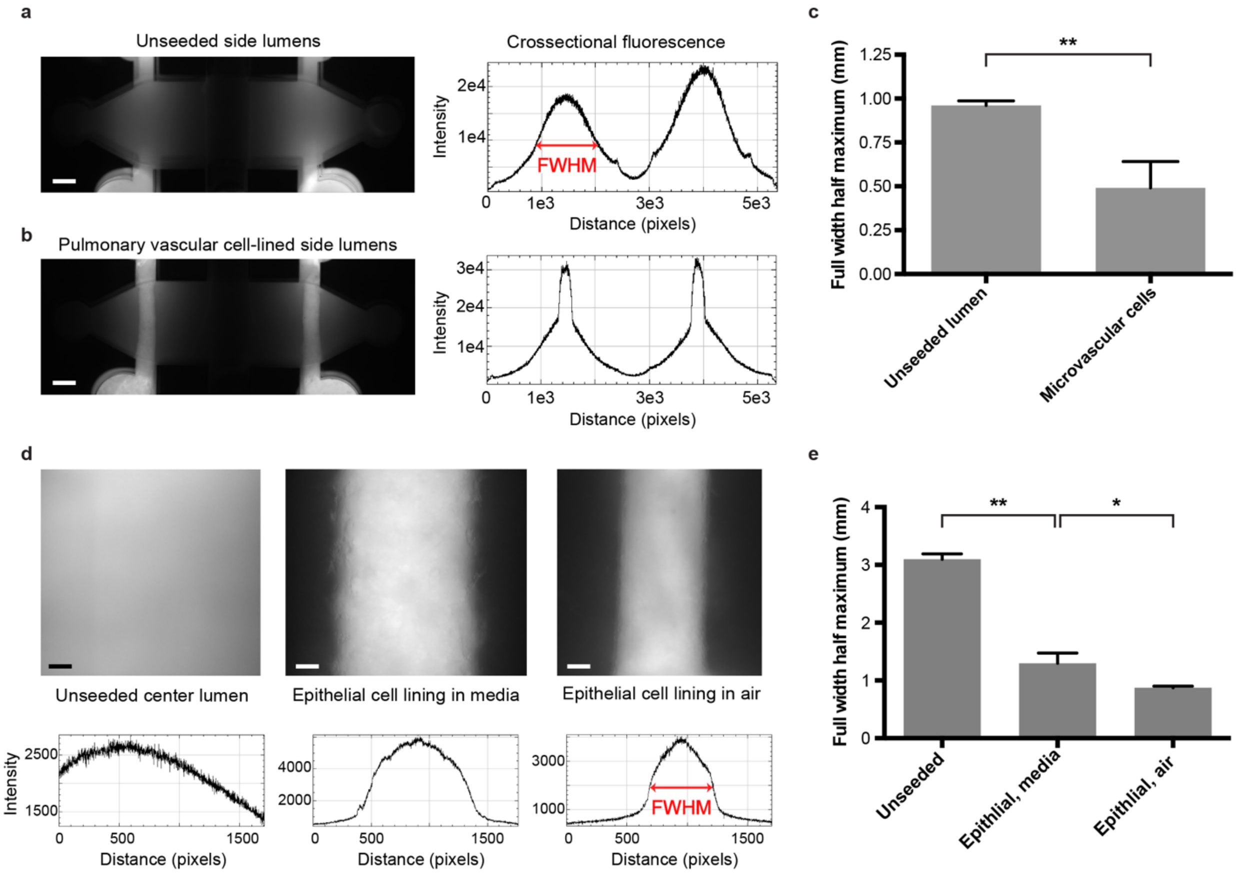

Supplementary Figure 2. Barrier function of cell monolayers in lumens. (a) Images of unseeded side lumens and (b) LMVEC-lined side lumens filled with 4 kD FITC dextran and measures of their crossectional fluorescence intensity values, with a depiction of the full width at half maximum (FWHM) metric. (c) FWHM values for four side lumens of conditions shown in (a, b). (d) Images of center lumens that are unseeded, seeded with HBECs for six days in growth media, BEGM, and seeded with HBECs for two days in BEGM, one day in differentiation medium, and one day exposed to air. (e) Mean FWHM values for three lumens of each condition in (d). Error bars represent standard deviation. Conditions in (c) and (e) were compared using unpaired, two-tailed Student's t-tests. * p-value < 0.05, ** p-value < 0.01. All scale bars are 250 μm.

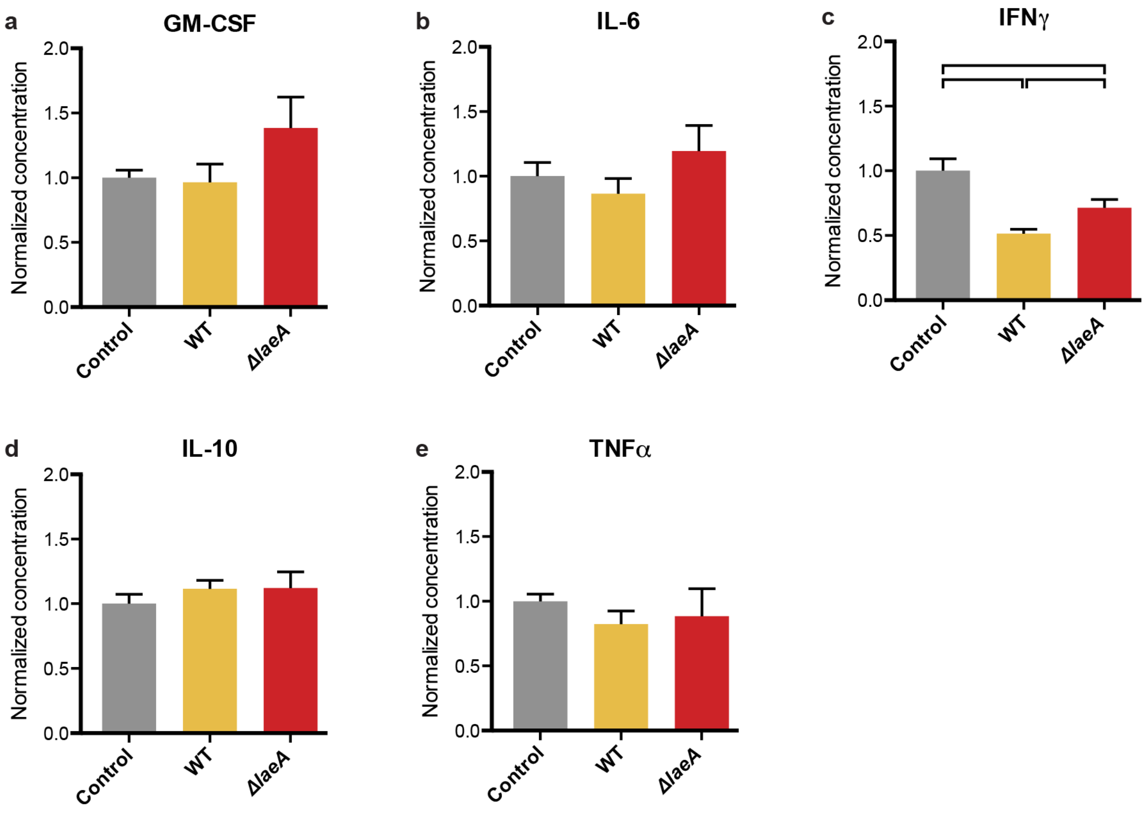

Supplementary Figure 3. Direct fungal contact stimulates cytokine response in the organotypic bronchiole model. (a-e) Cytokine concentrations measured in media harvested from the side, endothelial cell-lined lumens of bronchiole models inoculated with WT *A. fumigatus* spores or *ΔlaeA A. fumigatus* spores normalized to control, non-inoculated bronchiole models. Each plotted bar represents the mean of nine organotypic devices prepared on three separate days and error bars represent standard error of the mean. Data were analysed using one-way ANOVA with Tukey's multiple comparisons test and horizontal brackets denote comparisons between conditions that are statistically significant (p-value < 0.05). Additional cytokines from this experiment are presented in Fig 2.

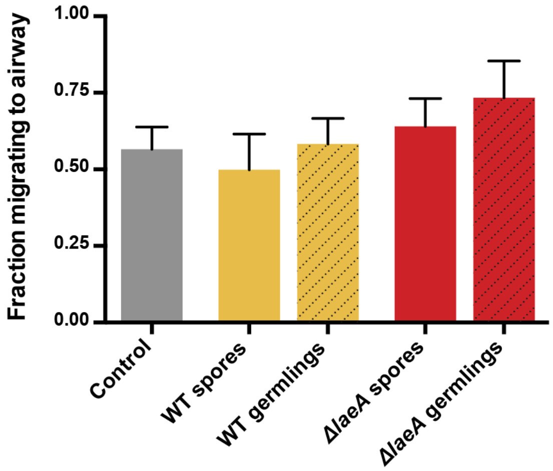

Supplementary Figure 4. PMN migration in models of early fungal infection is limited. Plotted are the fractions of PMNs that extravasated from the airway-facing side of the endothelial lumens and migrated in the direction of the center, hyphae-invaded airway lumen, compared with those that extravasated and migrated in the opposite direction. Each plotted bar represents the mean of six side lumens in three organotypic devices and error bars represent standard error of the mean. Data were analysed using one-way ANOVA with Tukey's multiple comparisons test and none of the pairwise comparisons are statistically significant to p-value < 0.05.

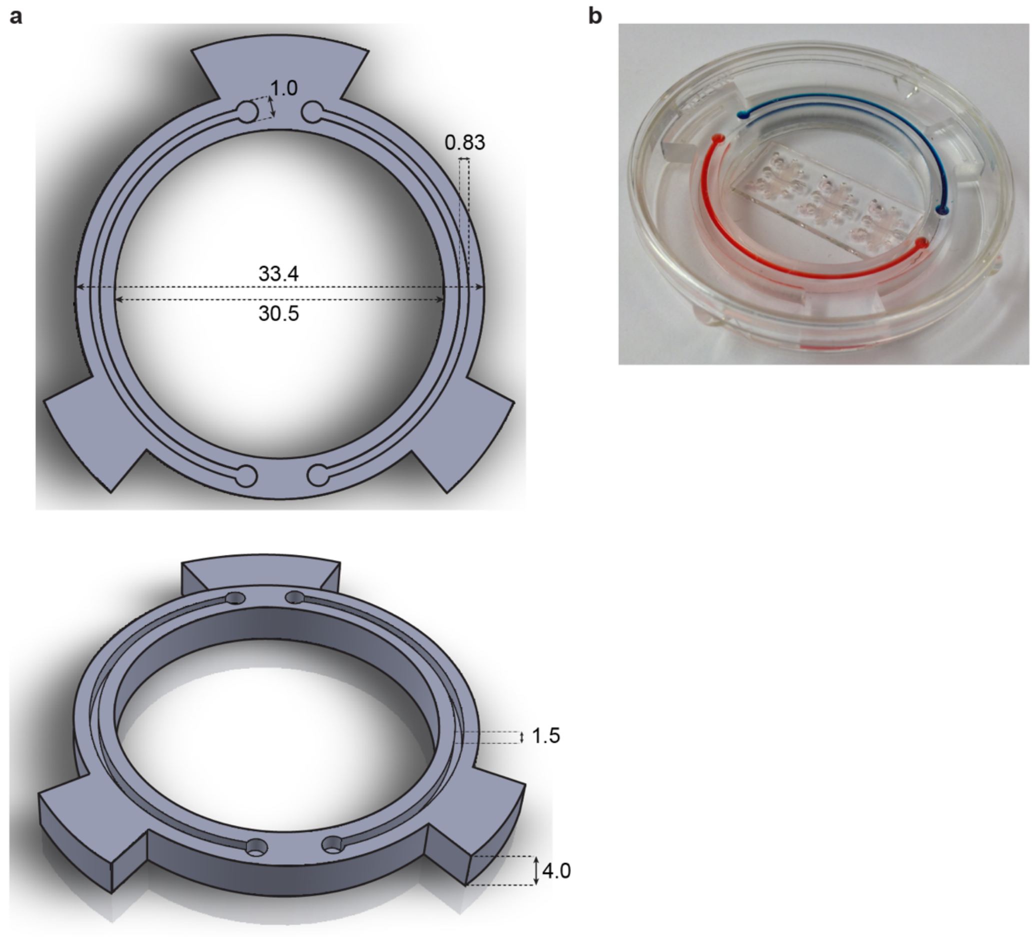

Supplementary Figure 5. Microbial culture insert schematic and photo. (a) Dimensions of the culture dish insert. All measurements are given in mm. (b) Experimental setup. Three organotypic bronchiole devices occupy the center of a 50 mm glass-bottom dish while a plastic dish insert contains microbial culture channels, as visualized with red or blue dye, on either side. A secure lid allows for volatile communication between the microbial cultures in the insert and the three center lumens exposed to air.

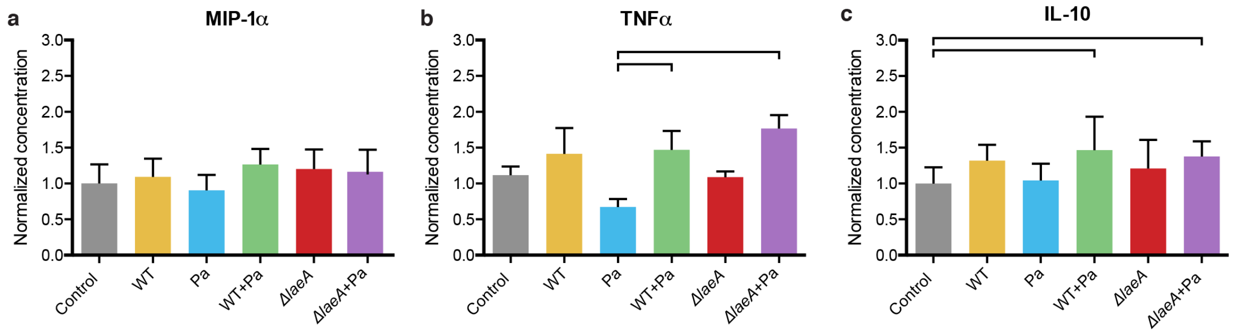

Supplementary Figure 6. Microbial volatiles impact epithelial cell secretion of inflammatory cytokines. (a-c) Cytokine levels in conditioned media from the side, endothelial-lined lumens measured after 24 h of volatile coculture with an insert all non-inoculated, half WT *A. fumigatus*, half *P. aeruginosa*, half each WT *A. fumigatus* and *P. aeruginosa*, half *ΔlaeA A. fumigatus*, and half each *ΔlaeA A. fumigatus* and *P. aeruginosa*. Each plotted bar represents the mean of nine organotypic devices prepared on three separate days and error bars represent standard error of the mean. Data were analysed using one-way ANOVA with Tukey's multiple comparisons test. Horizontal brackets denote comparisons between conditions that are statistically significant (p-value < 0.05). Additional cytokines from this experiment are presented in Fig 4.
