# Supplementary material for: Microbial volatile communication in human organotypic lung models
Source: Nat Commun. 2017 Nov 24;8:1770. doi: 10.1038/s41467-017-01985-4 (PMC5701243; doi:10.1038/s41467-017-01985-4)
Supplement: Supplementary file 3 — Description of Additional Supplementary Files [file 41467_2017_1985_MOESM3_ESM.pdf]

Description of Additional Supplementary Files

File Name: Supplementary Movie 1
Description: Movie of PMN extravasation and migration toward fungal hyphae. WT condition from Figure 4. Interval between frames is 5 minutes.

File Name: Supplementary Movie 2
Description: Movie of PMN extravasation and migration toward fungal hyphae. WT condition from Figure 4. Interval between frames is 5 minutes.

File Name: Supplementary Data 1
Description: Editable .pdf file of the masks used to create organotypic bronchiole device master molds using soft lithography. All dimensions are to scale. Each layer is 350 µm thick when made into a master mold.

File Name: Supplementary Data 2
Description: Solidworks file for the microbial culture insert.
