# Supplementary figures and images for: Microbial volatile communication in human organotypic lung models
Source: Nat Commun. 2017 Nov 24;8:1770. doi: 10.1038/s41467-017-01985-4 (PMC5701243; doi:10.1038/s41467-017-01985-4)

Master 1: bottom half of device

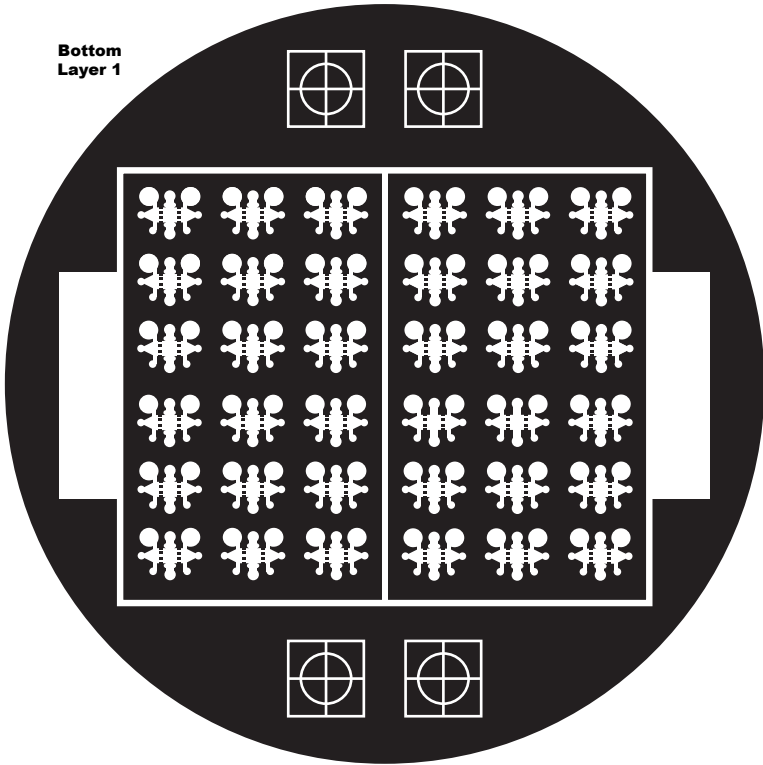

Master 2: top half of device

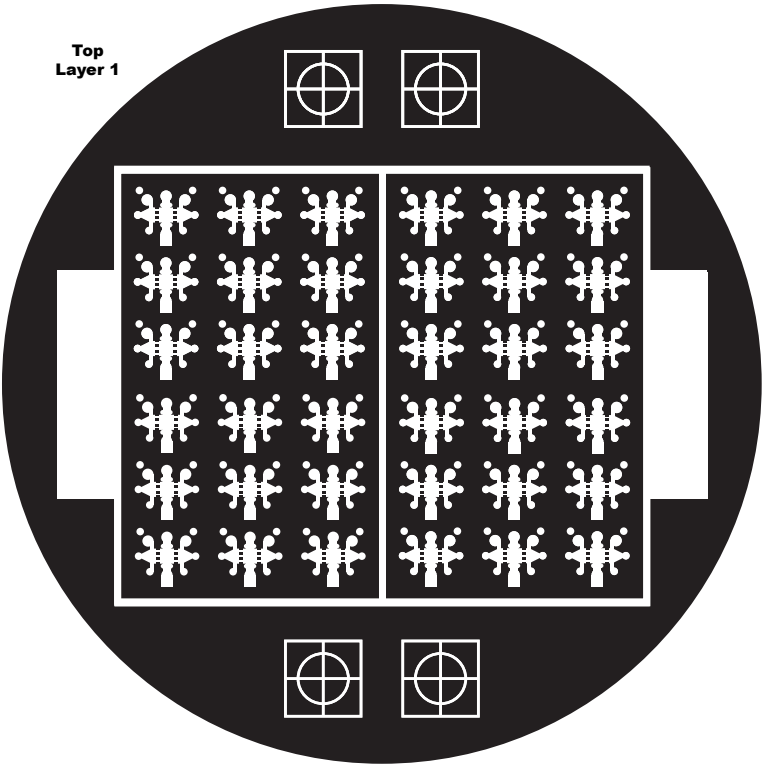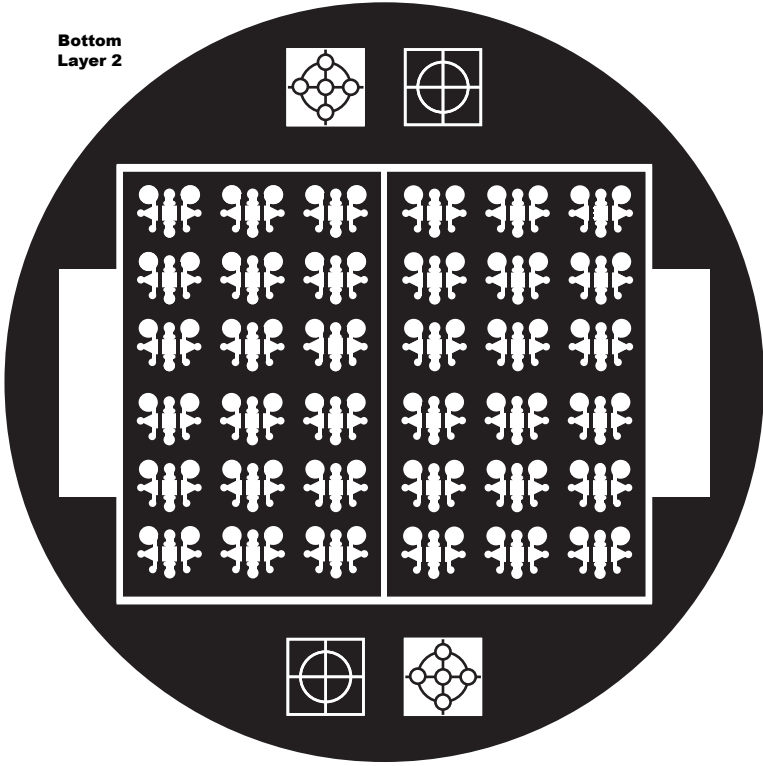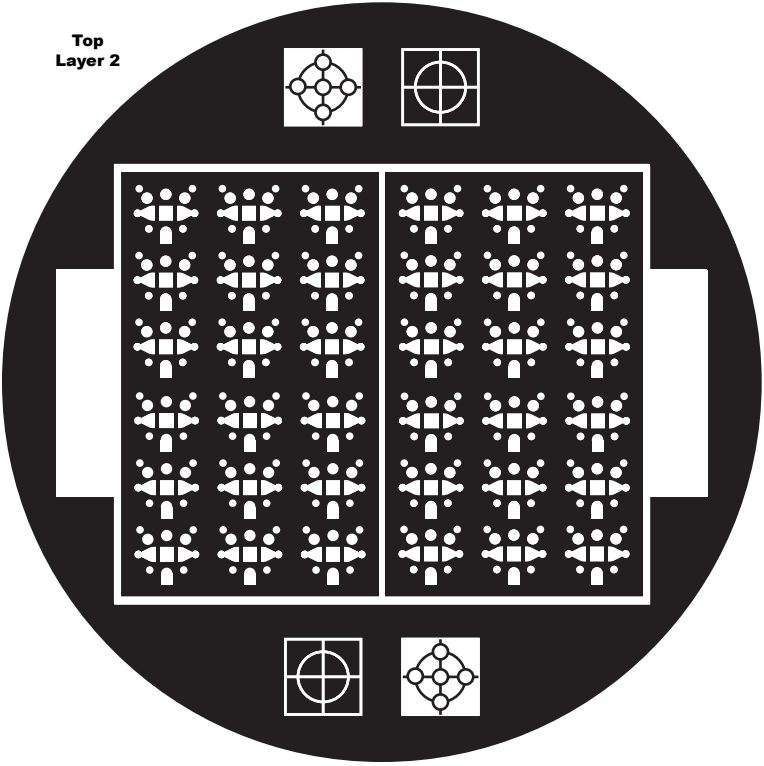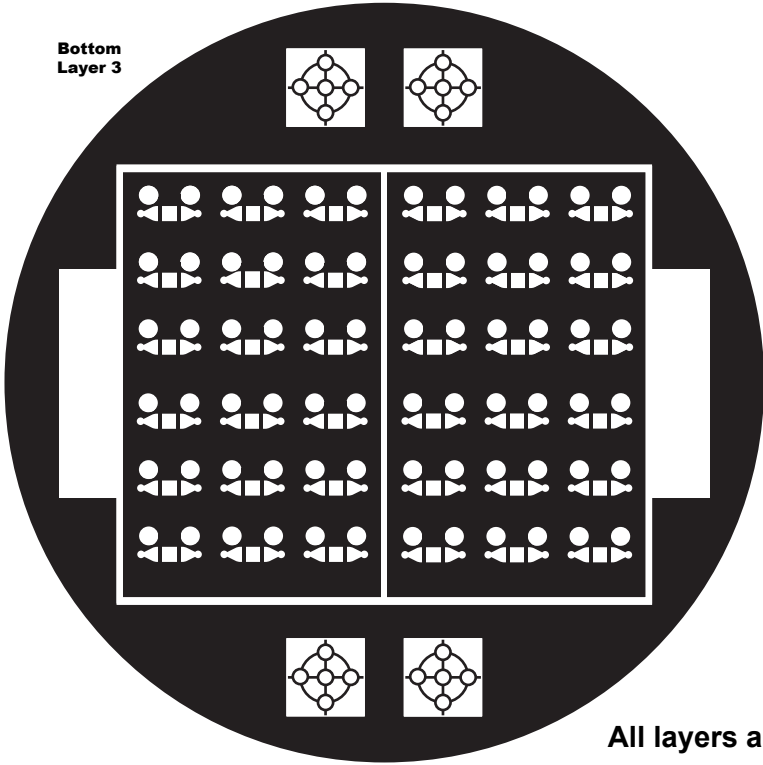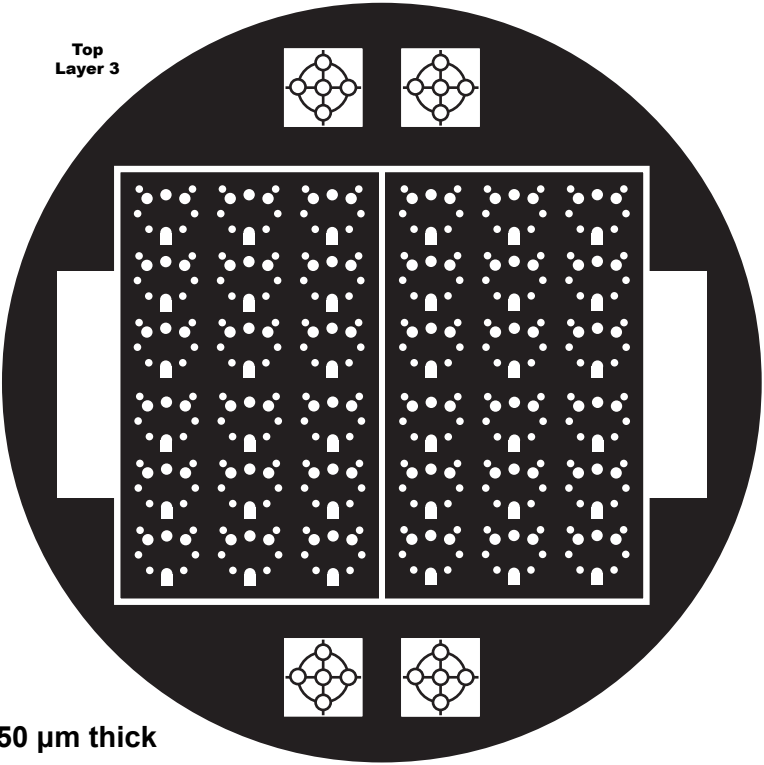

All layers are 350  $\mu$ m thick

Supplement: Supplementary file 4 — Supplementary Data 1 [file 41467_2017_1985_MOESM4_ESM.pdf]
